# Bicuspid aortic valve; optimal diagnosis and latest interventional treatment

**DOI:** 10.1007/s12471-015-0649-x

**Published:** 2015-01-28

**Authors:** Ernst E. van der Wall

**Affiliations:** Holland Heart House/Netherlands Society of Cardiology, Moreelsepark 1, 3511 EP Utrecht, The Netherlands

Bicuspid aortic valve (BAV) is one of the most common congenital heart defects with a population prevalence of 0.5 to 1.3 % [1]. The defect is considered to be a heritable disorder with a family recurrence rate of approximately 35 %. Recent studies show that mutations in the NOTCH1 gene are associated with BAV [2]. BAV progresses more rapidly into regurgitation or stenosis of the valve [3]. This results in a higher occurrence of aortic valve replacement, especially at younger age. Additionally, BAV is more susceptible than a tricuspid aortic valve (TAV) to nest bacteria or other organisms, leading to endocarditis. BAV is not only a peculiar valve morphology leading to specific valve pathology, it is also frequently associated with (asymptomatic) ascending aorta dilatation which leads to an increased susceptibility to ascending aortic aneurysms and aortic dissection [4]. Aortic elasticity measurements of BAV patients suggest that diminished aortic elasticity is at least part of its causation.

## Optimal diagnosis

Patients with BAV frequently remain undiagnosed until the manifestation of symptoms. Therefore, early screening and detection of patients is warranted. Imaging of a severely stenotic aortic valve is challenging. Due to the severity of stenosis and calcified nature of the aortic valves, echocardiography is frequently unable to differentiate between TAV and BAV. In a previous study published in the Netherlands Heart Journal in 2011 [5], cardiovascular magnetic resonance (CMR) was able to assess aortic valve morphology in BAV patients more frequently than echocardiography (96 versus 73 %); CMR appeared to be more sensitive for detecting of BAV whereas echocardiography appeared to be more specific. Among unselected patients with severe aortic valve stenosis, a high percentage of patients with BAV was found (40 %). Patients with BAV are significantly younger and more frequently male. Typically, the ascending aorta was larger in patients with BAV. From a cost-effectiveness perspective, echocardiography will still be the first choice in BAV patients. When the echocardiograms are difficult to analyse or when in doubt, CMR can be useful to come to a diagnosis. In a recent study, CMR was found to be superior to transthoracic echocardiography for imaging of the aorta in patients with congenital aortic stenosis and BAV, especially at the level of the proximal ascending aorta when an aortic aneurysm is present [6]. In particular, when the ascending aorta appears large at echocardiography, it is important to evaluate progression of the aortic diameters with CMR as standard care [7, 8]. At present, multi-slice computed tomography (MSCT) is increasingly used for sizing TAV and BAV through noninvasive evaluation of the aortic root [9–11]. However, the true gold standard for assessing these stenotic valves appears to be the appraisal of the surgeon.

## Latest interventional treatment

Currently, BAV stenosis and/or regurgitation is the most common indication for surgical aortic valve replacement in patients < 70 years of age. Nonetheless, 20 % of patients > 80 years of age have underlying bicuspid pathology. Over the past 10 years, transcatheter aortic valve replacement (TAVR) has become a standard procedure in elderly patients with severe inoperable aortic stenosis [12]. Recently, a study by Mylotte et al., published in JACC [13], evaluated the clinical value of TAVR in 139 BAV patients (mean age 78.0 ± 8.9 years) from 12 centres in Europe and Canada, being the largest collection of BAV patients treated with TAVR. Evaluation of the morphology of the aortic valve was performed using transoesophageal echocardiography in all patients; MSCT-based TAV sizing was used in 63.5 % of patients. Thirty-day device safety, success, and efficacy were noted in 79.1, 89.9, and 84.9 % of patients, respectively. There was a 30-day mortality rate of 5 %, a 30-day stoke rate of 2 %, and a device success rate of 90 %. One-year mortality was 17.5 %, and the patients were New York Hear Association functional class I or II. It was concluded that TAV-in-BAV is feasible with encouraging short- and intermediate-term clinical outcomes. However, a high incidence of post-implantation aortic regurgitation was observed of 28 %, which appears to be mitigated by MSCT-based TAV sizing (17 %). Since MSCT-based TAV sizing was clearly associated with reduced para-valvular regurgitation, MSCT should be considered a mandatory element of patient screening for TAV-in-BAV, certainly in view of the suboptimal echocardiographic results. In an accompanying Editorial by Colombo and Latib [14], it was stated that the incidence of significant aortic regurgitation, even with full MSCT evaluation, is still too high to extend TAVR to BAV unless the patient is truly inoperable or has an unacceptably high surgical risk. On the other hand, the Editorial reports that the current study sets a benchmark for next-generation TAVR devices demonstrating the feasibility of TAV-in-BAV.

To summarise, to diagnose patients with BAV, echocardiography remains the first choice. However, when the echocardiograms are difficult to analyse or for careful evaluation of the progression of aortic diameters, CMR is very useful to come to a diagnosis. MSCT is increasingly being used to accurately size the aortic root diameters. The recent study by Mylotte et al. [12] is the first large multicentre analysis of TAV implantation in patients with significant BAV stenosis or regurgitation. TAV-in-BAV proved to be feasible with encouraging short- and intermediate-term clinical outcomes, but the relatively high incidence (28 %) of post-implantation aortic regurgitation is of serious concern. Therefore, longer-term follow-up of a larger cohort of patients is required to more completely assess the efficacy and durability of TAV implantation in patients with bicuspid disease.
